# Capacitively Coupled Phase-based Dielectric Spectroscopy Tomography

**DOI:** 10.1038/s41598-018-35904-4

**Published:** 2018-12-03

**Authors:** Yandan Jiang, Manuchehr Soleimani

**Affiliations:** 1 Engineering Tomography Laboratory (ETL), Department of Electronic and Electrical Engineering, University of Bath, Claverton Down, BA2 7AY UK; 2 State Key Laboratory of Industrial Control Technology, College of Control Science and Engineering, Zhejiang University, Hangzhou, 310027 China

## Abstract

Impedance imaging is an effective approach for non-intrusively reconstruct the distribution of dielectric parameters inside the region of interest. Most common form of impedance imaging is electrical impedance tomography (EIT), which requires direct contact with the medium via electrodes. In this work we present a novel impedance imaging using capacitive coupling which provides a contactless method, totally non-invasive and non-intrusive, by measuring the phase. There are less attentions in many prior works to the phase information of the voltage/current measurements. This work studies for the first time the capacitively coupled electrical phase spectroscopy for contactless dielectric parameter imaging. A 12-electrode capacitively coupled test phantom and a measurement system were used to obtain the phase measurements within a wideband frequency range from 200 kHz to 15 MHz. Background data with different conductivity levels were investigated in the experiments to show a broad application possibility. The forward modelling was implemented by simulation and the image reconstruction based on phase measurements was implemented with the total variation algorithm. The potentials, possibilities and challenges of such capacitively coupled dielectric spectroscopy tomography with phase data are discussed in this work.

## Introduction

Electrical impedance tomography (EIT) is an important part in biomedical and industrial tomography due to its advantages of low cost, high speed and no hazard radiation^1^. Current research works and applications of EIT mainly focus on the real part or imaginary part of the measurement for separately conductivity or permittivity imaging^2–4^ and more importantly by direct contact of electrodes to the object. There are many issues associated with direct electrode contact. In cases where the two dielectric parameters are both changing, imaging of the conductivity or permittivity distribution separately may not work well because of the complex interplay of the two parts. Few research works pay attention to phase measurement, which involves both the two dielectric parameters.

To overcome the drawbacks resulted from the contact measurement principle of EIT, such as electrochemical erosion effect, polarization effect and contamination of the electrodes in industrial applications and electrode-skin contact impedance in medical applications, a lot of efforts have been made by researchers^5,6^. In 2013, the capacitively coupled contactless conductivity detection (C^4^D) technique was introduced to the EIT field and a capacitively coupled electrical resistance tomography was proposed^7,8^. By using two coupling capacitances, a measurement path can be developed for the AC excitation signal and detection signal can be obtained to implement contactless conductivity imaging^8^. This idea provides good references for other EIT-related research works.

This work aims to study capacitively coupled dielectric spectroscopy tomography based on phase measurement. First, a 12-electrode capacitively coupled EIT phantom was developed and the corresponding forward modelling was implemented by simulation. A sensitivity matrix which reveals the relationship between the dielectric parameter distribution and the phase measurement was obtained. Then, experiments were carried out with plastic rods of different sizes and conductive backgrounds of different conductivities to obtain the phase data within a wideband frequency range from 200 kHz to 15 MHz. Finally, image reconstruction was implemented by total variation (TV) algorithm on the basis of time-difference imaging, and phase-contrast spectroscopy imaging results were compared and discussed.

## Measurement Principle and Experimental Setup

**Figure 1:**
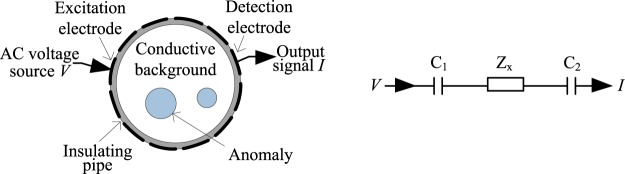
Construction of the capacitively coupled EIT phantom (**A**) and the equivalent circuit of a measurement electrode pair (B).

A capacitively coupled EIT phantom is developed for phase measurement, with 12 electrodes mounted equidistantly on the outer boundary of an insulating pipe. Figure 1(a) shows the construction of the phantom. One excitation electrode and one detection electrode will be selected to obtain one phase measurement, with the rest of the electrodes kept at floating potential. For the selected electrode pair, the two electrodes, the insulating pipe and the conductive background inside the phantom form two coupling capacitors C_1_ and C_2_. The medium in the sensing area can be equivalent to an impedance Z_x_. Figure 1(b) shows the equivalent circuit of a measurement electrode pair. So, the whole impedance *Z* obtained by a measurement electrode pair is

1 $$Z={Z}_{x}+\frac{1}{j\omega {C}_{c}}={Z}_{x}-j\frac{1}{2\pi f{C}_{c}}$$

where, *C*_*c*_ is the whole equivalent capacitance of the two coupling capacitances C_1_ and C_2_. *ω* and *f* are respectively the angular frequency and frequency of the excitation signal.

When an AC voltage is applied to the excitation, phase data of the output current signal can be obtained on the detection electrode. Numbering the electrode from 1 to 12, then 66 independent measurements can be obtained in a measurement cycle (i.e. excite electrode 1 and detect from electrode 2~12 one by one, then excite electrode 2 and detect electrode 3~12 by turn, …, finally excite electrode 11 and detect from electrode 12).

**Figure 2:**
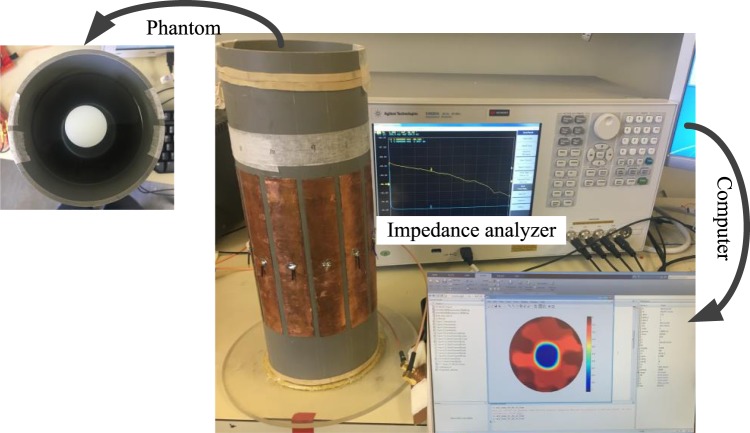
Measurement system.

Figure 2 shows the experimental setup of this work. An impedance analyzer is used to obtain the phase measurement with the developed phantom. A personal computer is used for data storage and image reconstruction. The inner and outer diameters of the phantom were 102 mm and 110 mm, respectively. The electrode angle and the electrode length of the phantom were 24° and 150 mm. The impedance analyzer Keysight 4990 A was used to stimulus the phantom and obtain phase measurements. The amplitude of the excitation voltage of the impedance analyzer was set to the maximum 1 V. The sweep frequency range was set from 10 kHz to 15 MHz.

## Methods

Forward modelling is an essential work in electrical tomography. The sensing region Ω of the phantom satisfies the Maxwell’s equations^9^

2 $$\nabla \times H=J+\,\frac{\partial D}{\partial t}$$

3 $$\nabla \times E=-\,\frac{\partial B}{\partial t}$$

4 $${\rm{\nabla }}\cdot B=0$$

5 $${\rm{\nabla }}\cdot D=\rho $$

where, *H* is the magnetic field strength and *E* is the electric field strength. *J* is the current density and *D* is the electrical displacement vector. *B* is the magnetic induction. *ρ* is the charge density.

Now introduce the relationship between field quantities and medium characteristics:

6 $$D=\varepsilon E$$

7 $$J=\sigma E$$

8 $$B=\mu H$$

where, *σ*, *ε* and *μ* are the conductivity, permittivity and magnetic permeability of the medium.

The highest investigated frequency of the excitation electrical signal is 15 MHz in this work, so the corresponding wavelength is still much larger than the phantom size, which means the sensing region of the phantom can be regarded as a quasi-static electric field and the coupling effect between electric field and magnetic field can be neglected^8–10^. So, the time-harmonic electric field now satisfies (equation (3) becomes)

9 $$\nabla \times E=0$$

10 $$E=-\,\phi $$

where, *φ* is the time-varying electrical potential of the quasi-static electric field. Combining equations (6), (7) and (2) and applying sinusoidal steady state voltage to the system, the electric field satisfies

11 $$\nabla \times H=(\sigma +j\omega \varepsilon )E$$

Besides, to simplify the model, the fringe effect caused by the finite electrode length is neglected and a 2D model is established. Thus, taking the divergence of equation (11), the sensing area Ω can be modelled as^8^

12 $$\nabla \cdot ((\sigma (x,y)+j\omega \varepsilon (x,y))\nabla \phi (x,y))=0\,(x,y)\subseteq {\rm{\Omega }}$$

where, *σ*(*x*, *y*), *ε*(*x*, *y*) and *φ*(*x*, *y*) are the spatial conductivity, permittivity and electrical potential distributions. The boundary conditions are defined as

13 $$\{\begin{array}{l}{\phi }_{a}(x,y)=V\\ {\phi }_{b}(x,y)=0\\ \partial {\phi }_{c}(x,y)/\partial \,\overrightarrow{n}=0\end{array}\,\begin{array}{r}(x,y)\subseteq {{\rm{\Gamma }}}_{a}\\ (x,y)\subseteq {{\rm{\Gamma }}}_{b}\,\\ (x,y)\subseteq {{\rm{\Gamma }}}_{c},\,(c\ne a,b)\end{array}$$

where, *V* is the amplitude of the excitation AC voltage source. Γ_1_, Γ_2_, Γ_3_, …, Γ_12_ represent the boundaries of the 12 electrodes. $\overrightarrow{n}$ denotes the outward unit normal vector. *a*, *b* and *c* are the indexes of the excitation electrode, the detection electrode and the floating electrodes, respectively.

A sensitivity matrix which reflects the relationship between the dielectric parameter and the phase measurement is required in solving the inverse problem (i.e. image reconstruction)^11^. This part is implemented firstly by the finite element method (FEM) for meshing the sensing area, then perturbation simulation of every element and finally post-processing to calculate the sensitivity matrix. First, the model in equations (12) and (13) is established with the software COMSOL Multiphysics and the sensing area is meshed into 1681 square elements as an essential part of FEM. Then, an AC voltage signal is applied to the excitation electrode *a* and the current measurement can be obtained on the detection electrode *b* by software, which is

14 $${{\rm{I}}}_{a-b}={\int }_{{{\rm{\Gamma }}}_{b}}\,{J}_{a-b}d({{\rm{\Gamma }}}_{b})$$

where, *a*-*b* is the measurement electrode pair. *J*_*a*-*b*_ is the current density measured on electrode *b* and Γ_*b*_ is the boundary of electrode *b*. Then the phase measurement of the output signal can be calculated

15 $${\theta }_{a-b}=\arctan ({\rm{Im}}({I}_{a-b})/\mathrm{Re}({I}_{a-b}))$$

With the phase measurement, the sensitivity matrix can be described as

16 $$S=[{s}_{ij}]$$

17 $${{\rm{s}}}_{ij}=\frac{\partial \theta }{\partial P}\approx \frac{{\theta }_{i}^{j}-{\theta }_{i}^{0}}{{P}^{1}-{P}^{0}}$$

where, *s*_*ij*_ is the sensitivity of the *j*th element under the *i*th electrode pair (i.e. the *i*th independent measurement, *i* = 1, 2, …, 66, *j* = 1, 2, …, 1681). *P* is the selected dielectric parameter, either the conductivity or the permittivity, and in some cases the combination of the two parameters (i.e. amplitude of the complex permittivity *ε*′ = *ε* + *jω𝜎*, where *ε* and *𝜎* are the permittivity and conductivity). ${\theta }_{i}^{0}$ represent the *i*th phase measurement when the pipe is full of conductive background (*P* = *P*^0^) and ${\theta }_{i}^{j}$ is the *i*th phase measurement when the dielectric parameters of the *j*th element chang from conductive background to anomaly (*P* = *P*^1^) and the remaining elements are still kept at the dielectric parameters of the background (*P* = *P*^0^).

Image reconstruction is to determine the dielectric distribution Δ*P* from the phase projection vector Δ*θ*, based on the pre-determined sensitivity matrix *S*, which means solving the following equation^12,13^

18 $${\rm{\Delta }}\theta =S{\rm{\Delta }}P$$

where, Δ*θ* is the time-difference phase projection vector obtained by practical phase measurements and Δ*P* is the relative dielectric parameter distribution inside the sensing area.

The imaging part is based on the commonly used time-difference imaging method^14^, where two phase measurements are obtained at different times: one is taken as a reference when the phantom is full of homogeneous conductive background and the other is taken when the anomaly/object is introduced into the phantom. And the time-difference phase projection is the difference of the two phase measurements.

The general and simplest way to solve equation (18) is introducing the least-squares method and minimizing the sum of square errors

19 $${\rm{\arg }}\,{{\rm{\min }}}_{{\rm{\Delta }}P}\,\frac{1}{2}\parallel S{\rm{\Delta }}P-{\rm{\Delta }}\theta {\parallel }^{2}$$

Since the inverse problem is badly ill-posed because of the soft-field characteristic of EIT, a regularization penalty term *G*(Δ*P*) is applied to equation (19) and leads to the following optimization problem

20 $${\rm{\Delta }}P={\rm{\arg }}\,{{\rm{\min }}}_{{\rm{\Delta }}P}\,\frac{1}{2}\,\parallel S{\rm{\Delta }}P-{\rm{\Delta }}\theta {\parallel }^{2}+G({\rm{\Delta }}P)$$

In this work, the *l*_1_-norm penalty term is introduced and the above inverse problem is solved by the total variation (TV) algorithm^15–17^ to obtain images which reflect the distribution of the dielectric parameter in the sensing area. The *l*_1_-norm penalty term of TV algorithm is

21 $$G({\rm{\Delta }}P)=\alpha \parallel \nabla {\rm{\Delta }}P{\parallel }_{1}$$

where, 𝛼 is the regularization parameter, $\nabla$ is the gradient and ||·||_1_ is the *l*_1_-norm.

Compared with *l*_2_-norm regularization (i.e. quadratic regularization, such as the widely used Tikhonov regularization), which tends to give “smoothed” or “blurred” images because the regularization gives strong penalty to edges, the *l*_1_-norm regularization works well to preserve intrinsic edges in original images and has good resistance to data outliers and noises^18^. However, the objective function of TV regularization (equations (20) and (21)) can not be effectively solved by traditional linearization techniques because it is non-differential. For this problem, previous research works have verified that the split Bregman (SB) iterative algorithm was effective to split the data fidelity term and the non-differential *l*_1_-norm penalty term of the objective function to a sequence of unconstrained problems which can be easily solved^19^. So, the SB method is used in this work. More details concerning the SB-based TV algorithm are available in reference^15–19^.

## Results

With the measurement system, experiments were carried out using saline (as backgrounds) and plastic rods (as anomalies). Different sizes and positions of the anomaly were investigated and the influence of the background conductivity was studied as well. The conductivity of the background in the phantom was measured by the Jenway 4510 conductivity meter and the experimental temperature was around 23.8 degrees Celsius. Spectroscopy imaging results of the following frequencies (200 kHz, 500 kHz, 1 MHz, 5 MHz, 6 MHz, 10 MHz and 15 MHz) are listed.

**Figure 3:**
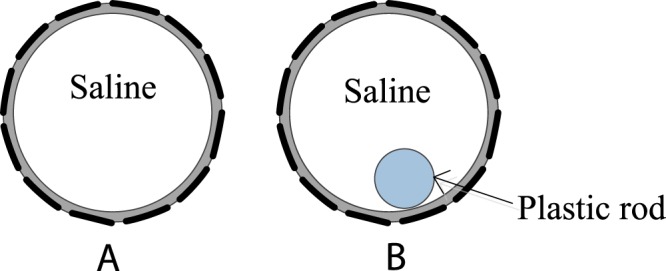
Setups for model verification: only saline background in the phantom (**A**) and a plastic rod (OD 31 mm) inside the background (**B**).

Before the main experiments and imaging work, it is important to verify the effectiveness of the developed mathematical model and the performance of the phantom. Both simulation work and experiments were undertaken to obtain wideband phase measurements. Simulation results are obtained with the established model and experimental results are obtained with the real phantom. Figure 3 shows the setup of only the saline background inside the phantom and the setup with a plastic rod (OD 31 mm) inside the background. The conductivity of the background is 0.065 S/m. Phase measurements of these two cases obtained by simulation and by experiments are compared. Besides, time-difference phase projections obtained with different background conductivities are compared as well.

**Figure 4:**
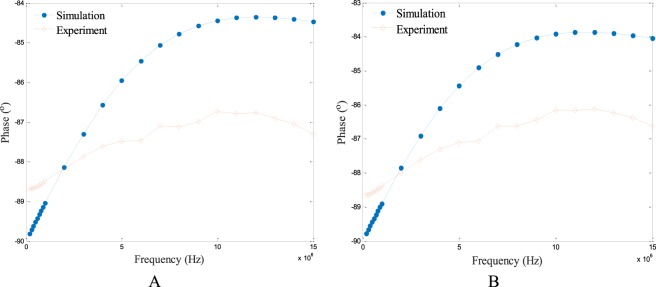
Spectroscopy phase measurements obtained by simulation and experiment for the two cases in Fig. 3: only background (**A**) and a plastic rod inside the background (**B**).

Figure 4A,B show the spectroscopy phase measurements obtained by both simulation and experiment of the two cases in Fig. 3, respectively. Every phase measurement in the curve is the mean value of 66 independent phase measurements obtained at a specified frequency. It can be seen that the spectroscopy phase measurements obtained by simulation and those obtained by experiment have good accordance. Besides, it is indicated that when the frequency is below 10 MHz, the phase measurements show an increasing trend as the frequency increases, while when the frequency goes higher than 10 MHz, the phase measurements show a gentle turning point and start to show a different trend. This curve illustrates the internal change of the electromagnetic field and the internal interplay of dielectric parameters.

**Figure 5:**
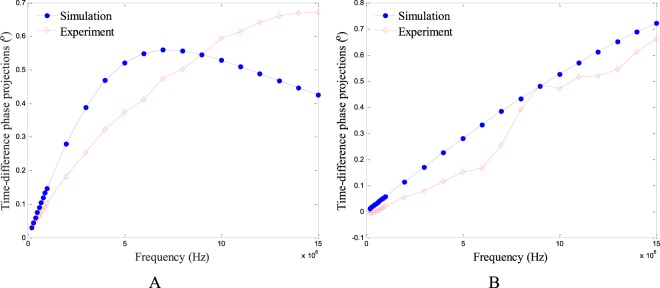
Spectroscopy time-difference phase projections obtained by simulation and experiment with the background conductivity of 0.065 S/m (**A**) and 0.3 S/m (**B**).

Figure 5 shows the relationship between the time-difference phase projections and the frequency at low-conductivity background (0.065 S/m) and high-conductivity background (0.3 S/m), respectively. The time-difference phase projections in Fig. 5 shows similar trends between simulation data and experimental data. And at high conductivity background, the time-difference phase data has good linearity, both for simulation and experiments.

**Figure 6:**
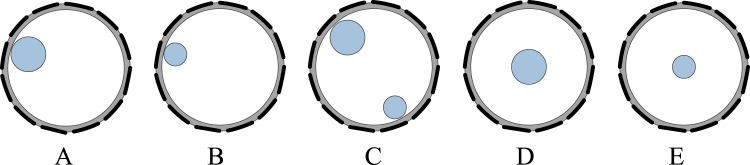
Five experimental setups: one plastic rod (OD 31 mm) positioned on the edge of the sensing area (**A**), one plastic rod (OD 21 mm) positioned on the edge of the sensing area (**B**), two plastic rods (OD 31 mm and OD 21 mm) positioned on the edge of the sensing area (**C**), one plastic rod (OD 31 mm) positioned in the centre of the sensing area (**D**), one plastic rod (OD 21 mm) positioned in the centre of the sensing area (**E**).

For the first part of experiments, five experimental setups formed by the two plastic rods were investigated using spectroscopy imaging with phase data. Figure 6A shows the experimental setup of the plastic rod (OD 31 mm) positioned on the edge of the sensing area and Fig. 6D shows the same plastic rod positioned in the centre of the sensing area. Figure 6B shows the experimental setup of the plastic rod (OD 21 mm) positioned on the edge of the sensing area and Fig. 6E shows the same plastic rod positioned in the centre of the sensing area. Figure 6C shows the two plastic rods placed at different positions on the edge of the sensing area.

**Figure 7:**
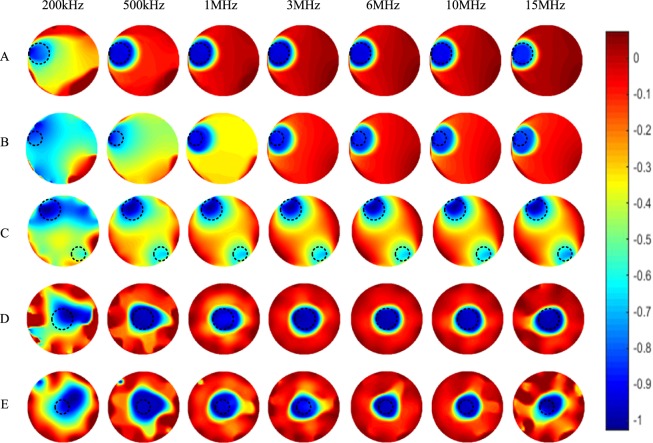
Spectroscopy imaging results of the five experimental setups in Fig. 6 with the background conductivity of 0.1 S/m.

Figure 7 shows the corresponding imaging results of the five experimental setups with the background conductivity of 0.1 S/m and a frequency range from 200 kHz to 15 MHz. The true positions and sizes of the plastic rods are marked in the images with black dashed circles. It is difficult to get clear image of the plastic rod inside the conductive saline background at low frequency (200 kHz). But when the frequency goes higher, clear images of the plastic rods on the edge of the phantom can be reconstructed. For two plastic rods inside the sensing area, relatively blurred images can be obtained. Besides, it is difficult to differentiate the size of the plastic rod positioned in the centre at low frequencies as the images of the two plastic rods (OD 31 mm and OD 21 mm) show similar sizes of the rods in the centre at frequencies below and including 1 MHz.

For the second part of experiments, the influence of background conductivities on imaging was studied. Experiments of five saline backgrounds with the conductivities of 0.065 S/m, 0.1 S/m, 0.2 S/m, 0.3 S/m and 0.4 S/m were carried out respectively.

**Figure 8:**
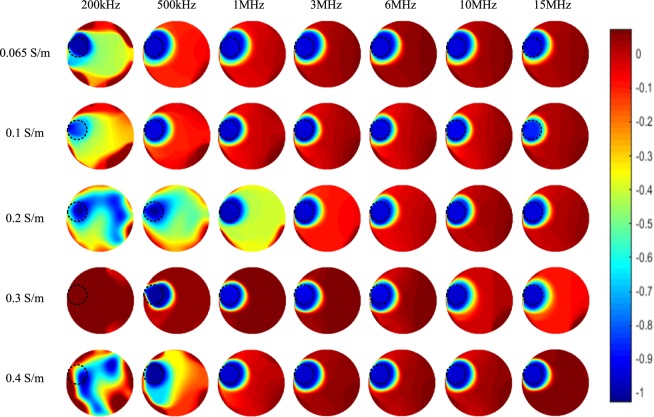
Spectroscopy imaging results of one plastic rod (OD 31 mm) positioned on the edge of the sensing area under five backgrounds with different conductivities.

Figure 8 shows the corresponding imaging results of one plastic rod (OD 31 mm) positioned on the edge of the sensing area (which is similar with the setup in Fig. 6A) under the five backgrounds. For backgrounds of low conductivities (i.e. 0.065 S/m and 0.1 S/m), images of the whole tested frequency range can reflect the true distribution in the sensing area. While for backgrounds of high conductivities (i.e. 0.2 S/m, 0.3 S/m, especially 0.4 S/m), images obtained at low frequencies are more vulnerable to noises and increasing the excitation frequency helps to improve the image quality. For all the spectroscopy imaging results in Fig. 8, it is indicated that higher excitation frequency can improve the overall signal to noise ratio of the images.

**Figure 9:**
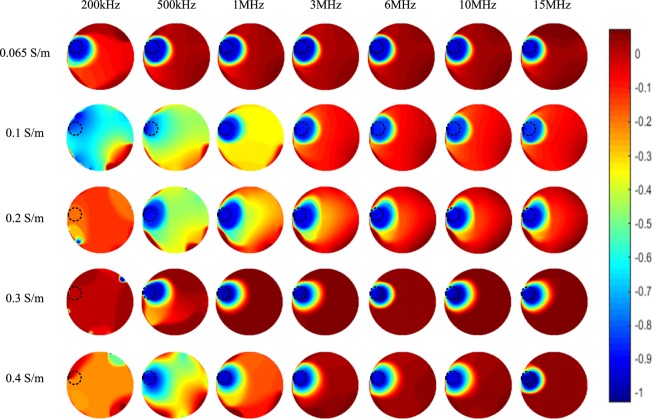
Spectroscopy imaging results of one plastic rod (OD 21 mm) positioned on the edge of the sensing area under five backgrounds with different conductivities.

Figure 9 shows the imaging results of a smaller-sized plastic rod (OD 21 mm) positioned on the edge of the sensing area (which is similar with the setup in Fig. 6B) under the five backgrounds. Similarly, the images reconstructed at low frequencies are noisier than those reconstructed at high frequencies.

**Figure 10:**
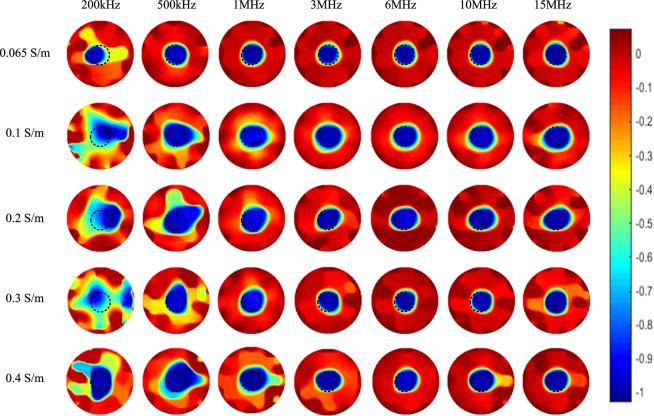
Spectroscopy imaging results of one plastic rod (OD 31 mm) positioned in the centre of the sensing area under five backgrounds with different conductivities.

Figure 10 shows the imaging results of one plastic rod (OD 31 mm) positioned in the centre of the sensing area, i.e. the setup in Fig. 6D. It is inspiring to find that imaging of the central area in the sensing area based on the phase data has similar performance with that of the near-edge area, although the sensitivity near the pipe wall is much higher than that in the centre. For all background conductivities, images reflect the exact position and size of the plastic rod can be obtained when the frequency is higher than 1 MHz. Within the tested conductivity range of the background, the results show that the background conductivity is not an important limitation of phase measurement, which indicates more broad applications potential of this method.

## Discussion

This work investigates the capacitively coupled dielectric spectroscopy imaging based on wideband phase data. From the above results, it can be found that for general applications (within a range of the background conductivity from 0.065 S/m to 0.4 S/m), the phase-based spectroscopy imaging performs well and can obtain good images for most frequencies in the range from 500 kHz to 15 MHz. Combining all the images in Figs 7–10, the images obtained at 3 MHz and 6 MHz have the best overall performance. At low frequency, i.e. 200 kHz or even lower, the plastic rod can not be reconstructed accurately due to the high equivalent impedance of the coupling capacitances at low frequency, which is clearly indicated in equation (1). For high background conductivity (higher than 0.3 S/m), increasing the frequency within a reasonable range can not only decrease the equivalent impedance of the coupling capacitances, but also reduce the noise level of reconstructed background. So, better images of the dielectric distribution can be obtained at high frequency. It is necessary to note that the excitation AC voltage signal is provided by the impedance analyser, whose maximum amplitude is 1 V. This signal is a bit small to get high-SNR phase data and the corresponding images. Excitation signal with higher amplitude may help to get better phase data and clearer images, especially for lower frequency.

For the high frequency cases where the frequency is higher than 10 MHz, a slight shrink in the size of the reconstructed anomaly positioned on the edge can be observed. More attentions to be paid when dealing with higher conductivity background and higher frequency excitation. When the frequency or conductivity is high enough, the skin effect should be taken into consideration. The skin depth *δ* in ‘Antenna Theory’ is defined as^20^

22 $${\boldsymbol{\delta }}=\sqrt{\frac{1}{{\boldsymbol{\pi }}f{\boldsymbol{\sigma }}{\boldsymbol{\mu }}}}$$

where, *𝜎* is the conductivity of the conductive medium, *f* is the frequency of the excitation signal, *μ* is the permeability of the conductive medium.

In this work, the highest frequency and background conductivity investigated are 15 MHz and 0.4 S/m, respectively. According to equation (22), the skin depth in this case is approximately 205 mm, which is larger than the phantom size (outer diameter of 110 mm) and far from causing obvious skin effect. So, skin effect is not the reason of size shrinkage. Concerning the model in equation (12), the corresponding wavelength of 15 MHz excitation signal is about 20 m, which is still much larger than the size of the phantom and the inclusion. That means the sensing area can still be regarded as quasi-static electric field and the mathematical model in equation (12) can still be used theoretically. Combining the mentioned two points, the possible explanation of the slight size shrinkage is that at high frequency (usually above 10 MHz), although the simplified Maxwell’s model for quasi-static electric field can still be used, it becomes less accurate. As a result, using the same sensitivity matrix of the sensing area will cause slight influence on final imaging results. Image degradation as the anomaly moves to the centre is not significant in our frequency range. However, for experiments with higher frequency range or more conductive mediums, full Maxwell’s equations will need to be considered in the forward modelling for future studies.

## Conclusion

For capacitively coupled phase measurement, experience and knowledge are very limited. This paper for the first time demonstrates capacitively coupled phase-based tomography. In addition to design specified measurement system (excitation signal and data acquisition) and investigate the modelling in different frequency range, phantom optimization (material of the pipe, size of the electrode) should be considered to further enhance the performance of phase imaging. Besides, phase measurement is relatively sensitive to the environment as it involves the imaginary part. So, shielding methods should also be introduced as part of the optimization. Despite all the above challenging factors, very good imaging of samples in conductive medium was shown in experimental results in this study. Spectroscopy tomography images produced in this paper bridges an important gap in multi-dimensional sensing through a novel contactless approach, which otherwise is not available. We hope this study will open up many new applications for electrical imaging.
